# Temperature and the Field Stability of a Dengue Rapid Diagnostic Test in the Tropics

**DOI:** 10.4269/ajtmh.15-0142

**Published:** 2015-07-08

**Authors:** Koukeo Phommasone, Onanong Sengvilaipaseuth, Xavier de Lamballerie, Manivanh Vongsouvath, Ooyanong Phonemixay, Stuart D. Blacksell, Paul N. Newton, Audrey Dubot-Pérès

**Affiliations:** Microbiology Laboratory, Lao-Oxford-Mahosot Hospital-Wellcome Trust Research Unit (LOMWRU), Mahosot Hospital, Vientiane, Lao People's Democratic Republic; UMR_D 190 “Emergence des Pathologies Virales” (Aix-Marseille University, IRD French Institute of Research for Development, EHESP French School of Public Health), Marseille, France; Centre for Tropical Medicine and Global Health, Nuffield Department of Clinical Medicine, University of Oxford, Churchill Hospital, Oxford, United Kingdom; Mahidol-Oxford Tropical Medicine Research Unit, Faculty of Tropical Medicine, Mahidol University, Bangkok, Thailand

## Abstract

The global incidence of dengue has increased significantly in recent decades, resulting in a large public health burden in tropical and subtropical countries. Dengue rapid diagnostic tests (RDTs) can provide accurate, rapid accessible diagnosis for patient management and may be easily used by health workers in rural areas. However, in dengue-endemic areas, ambient temperatures are often higher than manufacturer's recommendation. We therefore evaluated the effect of high temperature over time on the performance of one commonly used dengue RDT, the Standard Diagnostics Bioline Dengue Duo. RDTs were kept in five different conditions (at 4°C, 35°C, 45°C, 60°C, and at fluctuant ambient temperatures in a free-standing hut) for between 2 days and 2 years in the Lao People's Democratic Republic (PDR). RDTs were tested with four control sera (negative, dengue nonstructural protein 1 [NS1], anti-dengue immunoglobulin [Ig] M, and anti-dengue IgG positive). The RDTs had 100% consistency over the 2-year study, despite high temperatures, including in the hut in which temperatures exceeded the manufacturer's recommendations for 29% of time points. These data suggest that the diagnostic accuracy of the SD Bioline Dengue Duo RDT remains stable even after long-term storage at high temperatures. Therefore, use at such ambient temperatures in tropical areas should not jeopardize the dengue diagnostic outcome.

## Introduction

The global incidence of dengue has increased significantly in recent decades. Over 2.5 billion people, nearly half of the world's population, are now at risk for contracting dengue, and 70% of those live in southeast Asia and the western Pacific.^1^ World Health Organization (WHO) estimates there may be 50–100 million dengue infections worldwide every year with an estimated 500,000 people requiring hospitalization with a 2.5% mortality.^1^ Using a cartographic approach, Bhatt and others^2^ estimated 390 million dengue infections per year, with only 96 million being symptomatic. The pathogen has also spread to new areas and is now endemic in more than 100 countries in Africa, the Americas, the eastern Mediterranean, southeast Asia, and the western Pacific.^1^ The presentation of the infection ranges from asymptomatic or flu-like illness to, occasionally, severe dengue and even death.

In the Lao PDR (Laos), urban areas such as the capital, Vientiane are particularly affected but there is also good evidence of dengue virus circulation in rural Laos.^3^–^5^ Dengue fever can be difficult to clinically distinguish from other common sympatric causes of fever in Laos such as scrub typhus, murine typhus, leptospirosis, typhoid, and Japanese encephalitis virus and chikungunya virus infections.^5^,^6^ Approximately one-third to two-thirds of patients clinically diagnosed with dengue do not have supportive laboratory etiological evidence.^7^–^9^

Laboratory techniques for dengue diagnosis, such as polymerase chain reaction (PCR) and enzyme-linked immunosorbent assay (ELISA) are expensive, requiring access to laboratory facilities with well-trained staff. In Laos, these techniques are available only in hospitals located in the capital city. An alternative diagnostic service for rural areas is immunochromatographic rapid diagnostic tests (RDTs). They are rapid, accurate, easy to use, and do not require specific technical knowledge or equipment.

The Standard Diagnostics (SD; Yongi, Gyeonggi, Republic of Korea) Bioline Dengue Duo RDT (SD dengue RDT) permits the concomitant detection of dengue nonstructural protein 1 (NS1) antigen (Ag) and anti-dengue immunoglobulin (Ig) M and IgG antibodies. Diagnostic evaluations that have compared ELISA, real-time reverse transcription (RT) PCR, virus isolation, and hemagglutination inhibition assay with the SD dengue RDT have demonstrated sensitivity and specificity higher than 80%.^10^–^13^ Therefore, the SD dengue RDT could be an appropriate test for dengue diagnosis in provincial hospitals and in the rural Lao context.

SD recommends RDT storage temperature between 1°C and 30°C. However, ambient temperatures in dengue-endemic countries are frequently higher. In Laos, where ambient temperature can exceed 40°C in the southern Mekong valley during the hot season, RDTs would need to remain accurate over wide range of conditions from air-conditioned laboratories to rural medical centers, and transportation, where electricity is not available or unreliable. There have been few investigations of the effect of high temperatures on the accuracy of RDTs. There has been great concern that the diagnostic accuracy of some commonly used malaria Ag-detection RDTs deteriorates through time at high-temperature storage.^14^–^18^ In response, Lon and others^19^ developed simple and inexpensive evaporative cooling boxes to reduce the temperatures malaria RDTs were exposed to in rural Cambodia.

In contrast, Learmonth and others^20^ found that six of seven human immunodeficiency virus (HIV) antibody (Ab) RDT kits were relatively robust, in terms of test accuracy, despite exposure to higher than recommended temperatures. Only one publication from Blacksell and others^21^ has previously reported the evaluation of the thermal stability of dengue RDTs. Eight commercial RDTs were kept in an incubator at 35°C over a period of 90 days; most of them were affected by heat with the sensitivity declining after 20 days except for the GlobaleMed (Alexandria, VA) dengue RDTs. However, the assays were limited in terms of duration and temperature and only IgM/IgG RDTs were tested. We therefore tested the stability of the SD Bioline Dengue Duo RDT (NS1 Ag and IgM/IgG) over 2 years at various temperatures in Laos, from 4°C to 60°C. We chose the SD RDT as it is commonly used in Laos.

## Methods

### Ethics statement.

Patients gave written informed consent to be included in an investigation of the causes of fever at Mahosot Hospital, Vientiane, Laos. For children, their guardians/parents gave informed written consent. This study was granted ethical approval by the Lao National Ethics Committee for Health Research and the Oxford Tropical Research Ethics Committee. (OXTREC No. 006-07). A dengue-naive individual gave written informed consent for his blood to be collected and then the serum to be used as negative control in this study.

### Description of RDT.

The SD dengue RDT is an in vitro immunochromatographic assay for the detection of dengue virus NS1 Ag and anti-dengue IgM/IgG antibodies in human serum, plasma, or whole blood, from finger-prick or venous blood. This test comprises a pair of test devices, a dengue NS1 Ag test on the left side, and a dengue IgM/IgG (Ab) test on the right side. Each device contains a nitrocellulose membrane strip enclosed in a plastic cassette. When a patient sample is loaded into the device, on the left strip, NS1, if present, binds to colloidal gold conjugated anti-NS1 antibodies. This complex migrates to the test band coated with anti-NS1 antibodies. On the right side, anti-dengue IgM and IgG, if present in patient serum, combine to dengue virus envelope proteins labeled with colloidal gold. The IgM and IgG complexes migrate up to two test bands where membranes are coated with anti-IgM complexes and anti-IgG complexes, respectively. The validity of the test is checked by the appearance of a control line on each strip. The test is easy to perform—three drops (using dropper provided with the kit, ∼100 μL) and 10 μL (using a capillary provided with the kit) of sample are applied into the two small wells on NS1 and Ab cassettes, respectively. Four drops of diluent (provided with the kit) are then applied on the Ab cassette. The test results are obtained in 15 minutes. The cassettes are enclosed within individual hermetically sealed foil pouches that are packaged in a set of 25 in cardboard boxes.

### RDT storage conditions.

**Figure 1.**
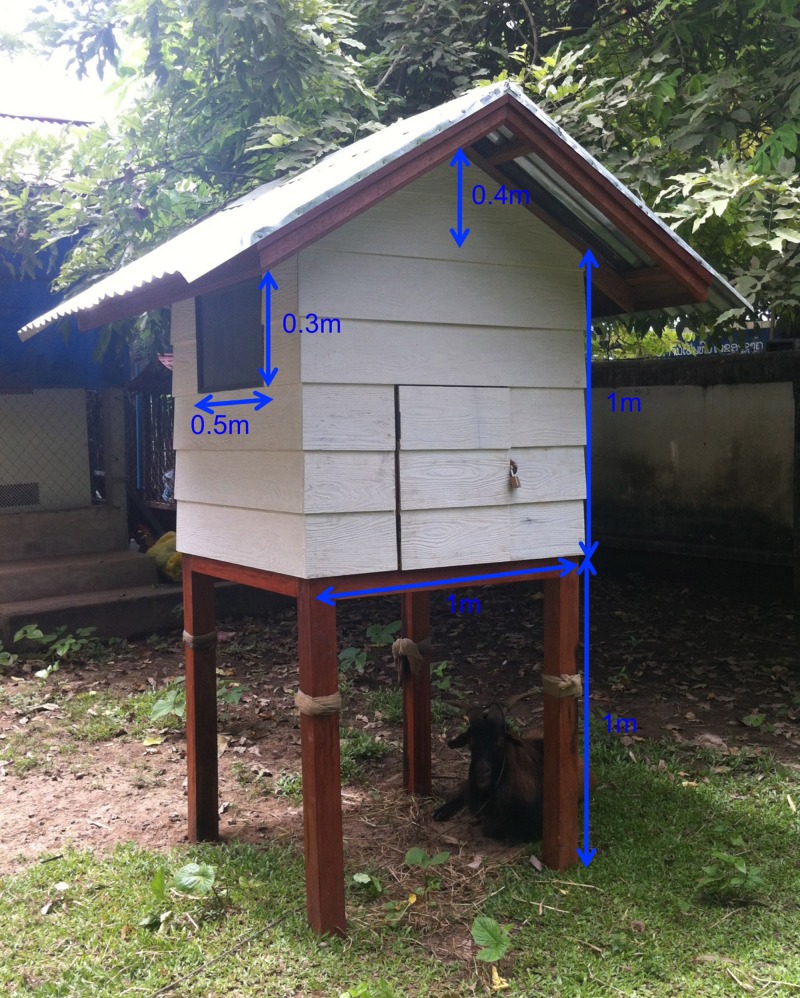
Hut for rapid diagnostic test (RDT) storage under field conditions. Hut constructed in wood to resemble miniature traditional Lao house. There is a goat beneath.

**Figure 2.**
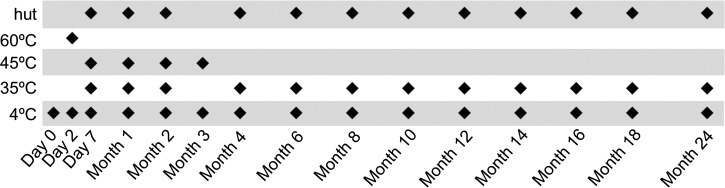
Time and temperature tested. Day 0 was July 23, 2012.

We tested extreme exposure in laboratory incubators at 60°C for 2 days and storage at 45°C over 3 months and at 35°C over 2 years, starting on July 23, 2012. Incubators were stabilized for 2 days at the required temperatures before adding the RDTs. To simulate storage condition in rural Laos, RDTs were placed in a small wooden hut in the garden outside the Microbiology Laboratory over 2 years (17.9592 N 102.6127 E, 172 m above mean sea level [msl]). This hut was constructed from wood as a miniature traditional Lao house (Figure 1

), to give an approximation of the temperatures experienced in a house in central Laos. The incubators provide evaluation at set temperatures only, while the use of the hut provides a real-life situation with temperature changing seasonally and diurnally. As reference, RDTs were also kept at approximately +4°C in a laboratory refrigerator. The RDTs were kept in their original packaging. The temperatures in all locations were recorded every 30 minutes using electronic thermometers (Tinytag Ultra 2 data logger [Gemini Data Loggers (UK) Ltd., Chichester, England], temperature resolution of 0.01°C and accuracy of approximately ±0.5°C) placed inside the RDT boxes. The RDTs in different storage conditions were tested according to the schedule shown in Figure 2

, in duplicate.

All RDTs were from the same manufacturer's lot (Lot No. 146001) and their expiry date was February 9, 2014, which means the study continued for 6 months after the expiry date.

### Preparation of control sera.

To evaluate the RDT performance over time and at differing temperature, duplicates of each sample were tested at each time/temperature point (Figure 2) with a total of 88 RDTs per sample tested. A minimum volume of 110 μL (100 μL for NS1 cassette and 10 μL for Ab cassette) was required to perform the RDT, so a minimum total volume of approximately 10 mL per sample tested was required (i.e., 110 μL × 88 = 9.68 mL). Because of the large volume of sample required and ethical restrictions, the use of a panel of individual patient sera was not possible or practical. Accordingly, since our objective was to detect a putative failure of the RDT for the detection of dengue virus NS1 and or anti-dengue IgM or IgG, we decided to use only four control sera: a negative control serum (negative for NS1, anti-dengue IgM, and IgG), an NS1 control serum (only positive for dengue NS1), an IgM control serum (only positive for anti-dengue IgM), and an IgG control serum (only positive for anti-dengue IgG). To be able to detect slight decreases in RDT performance, we wished to have control sera that were determined to be close to the limit of detection by RDT. To prepare these four control sera, we used patient sera, pooled when necessary, to reach the required volume and then diluted and tested to select the highest dilution positive by RDT.

Admission sera from dengue patients positive only for dengue virus NS1 or for anti-dengue IgM by SD RDT were selected to prepare NS1 and IgM control sera, respectively. Convalescent serum from dengue patient positive only for anti-dengue IgG by SD RDT was selected to prepare IgG control serum.

Serum from a dengue-naive patient was used as negative control serum and for the dilution of dengue patient sera for the preparation of the other control sera. Fifty aliquots of 250 μL of negative control serum (volume required to perform RDT in duplicate) were prepared and stored at −80°C.

**Figure 3.**
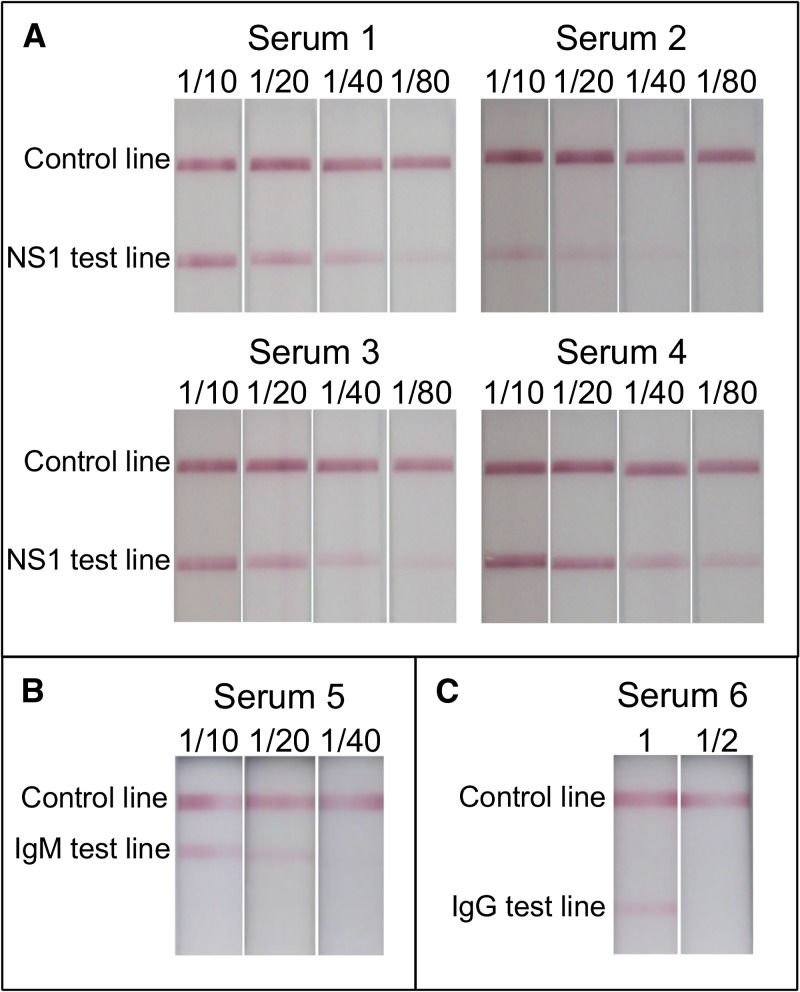
Rapid diagnostic test (RDT) results of patient serum dilutions for the preparation of control sera. (**A**) Nonstructural protein 1(NS1) strip result for NS1 control serum, (**B**) IgM/IgG strip result for IgM control serum, and (**C**) IgM/IgG strip result for IgG control serum.

To prepare NS1 control serum, 1/10, 1/20, 1/40, and 1/80 dilutions of four patient sera positive for NS1 (serum 1–4) were tested on RDT NS1 cassettes. The results displayed in Figure 3A show that 1/20 was the highest dilution that gave a clearly positive result by RDT with the volume being insufficient from a single patient. Accordingly, the sera from the four patients were pooled and diluted 1/20 to give a final volume of 13 mL. Fifty aliquots of 220 μL (i.e., the volume to perform the NS1 RDT in duplicate) of this NS1 control serum were prepared and stored at −80°C.

To prepare IgM control serum, 1/10, 1/20, and 1/40 dilutions of a serum from an anti-dengue IgM patient (serum 5) were tested by RDT. The results presented in Figure 3B show that 1/10 was the highest dilution giving a clear positive result. Then, 1.6 mL of dilution 1/10 of serum 5 was prepared and 50 aliquots of 25 μL (i.e., the volume to perform the Ab RDTs in duplicate) were prepared and stored at −80°C.

To prepare IgG control serum, 1/2 dilution of an anti-dengue IgG (serum 6) was tested by RDT and the results presented in Figure 3C showed that the dilution 1/2 was negative by RDT, which means that the non-diluted serum was already at limit of detection so was directly used as IgG control serum. Accordingly, serum 6 was aliquoted in 25 μL as previously described for IgM control serum.

### RDT testing and recording of results.

The RDT assays were performed following the manufacturer's instructions. To assure reproducibility, micropipettes, instead of the dropper and capillary provided with the kit, were used to apply the 100 μL and 10 μL of control sera on NS1 and Ab cassettes, respectively. Assay diluent used for dengue Ab test was kept in same locations as the dengue RDTs, which were taken out for testing following the schedule and then put back to the same location and temperature. At each time point (time and temperature), one aliquot of each control sera (NS1, IgM, IgG, and negative) was removed from freezer and tested in duplicate on two RDTs. To minimize inter- and intra-reader variability, two independent laboratory-trained investigators read all RDT results and were blinded to each other's results and the identity of the sera being tested one reader was unaware of the temperature storage conditions. The RDTs were read at exactly 15 minutes after sample addition.

## Results

### Temperature variability.

**Figure 4.**
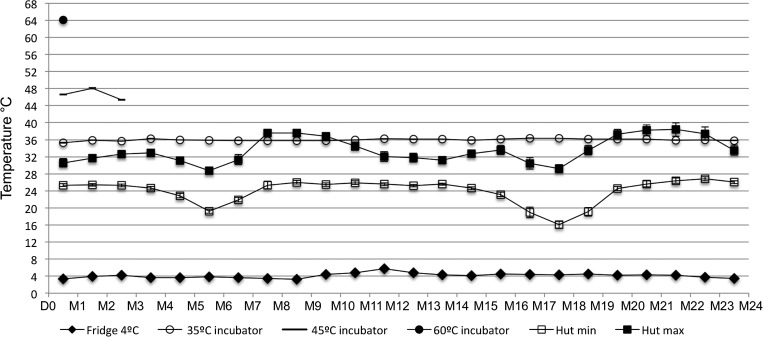
Monthly mean (95% confidence interval [CI]) temperatures recorded in the incubator, refrigerator, and hut over the 2-year study period, from July 23, 2012 to July 23, 2014. Empty squares are the average of daily minimum temperatures and black solid squares are the average of daily maximum temperatures recorded in the hut. D0 = day 0 (July 23, 2012), M = month.

Mean monthly temperatures, with 95% confidence interval (CI), recorded in the refrigerator (4°C), in the incubators (35°C, 45°C, and 60°C), and hut are presented in Figure 4.

#### Refrigerator (4°C).

The mean (95% CI) temperature within was 4.06°C (4.04–4.07°C). Sporadic elevation of the temperature was observed due to monthly cleaning of the refrigerator but this was never > 18°C and lasted a maximum of 30 minutes. Temperature data were missing for 9 days, as a consequence of the data logger being accidentally removed from the refrigerator.

#### In the incubators (35°C, 45°C, and 60°C).

Within the 60°C incubator for 48 hours, the mean (95% CI) temperature was 64.1°C (63.9–64.3°C), higher than the preset temperature by approximately 4°C. The temperature loggers in the 45°C and 35°C incubators for 90 days and 730 days, respectively, recorded mean (95% CI) temperatures of 46.6°C (46.5–46.7°C) and 36.0°C (36.0–36.0°C), respectively.

#### Temperature in the hut.

The temperature recorded in the hut was unavailable for 11 days as the storage capacity of the temperature logger was exceeded. Minimum and maximum temperatures recorded were 15.7°C and 42.0°C, respectively. The numbers of recordings for the five temperature groups are shown in Table 1. The RDTs kept in the hut were exposed to temperatures higher than 30°C, which is the RDT manufacturer's recommended maximum temperature, for 28.7% of time points during the 2-year study period. Temperatures exceeded 35°C for 6.7% of time points and even 40°C for 0.6% of time points in March, April, May, and early June.

### RDT results.

Inspection revealed no visual evidence of degradation of the RDT assay strips or diluent at different temperatures. Negative control serum was recorded as negative for all RDTs, no false positives were noted and no invalid tests occurred over the 2-year study period (Table 2).

Over the period of 24 months, 88 observations were made for NS1 detection using RDTs kept at 4°C, 35°C, 45°C, 60°C, and in the hut. The NS1 control serum was found positive for NS1 by all RDTs. There were no visually significant changes of intensity of the test and apparent control lines, compared with the reference RDTs (kept at 4°C). There was 100% concordance in the interpretation of the RDTs between the two readers. The IgM and IgG control sera were also respectively positive for IgM and IgG exclusively with no false positives with all RDTs tested over the 2-year period. The intensity of test lines were faint but this was found in all conditions, including the RDTs stored in the refrigerator, and was similar to line intensities observed during the preparation of positive control sera. This has no impact on the interpretation of the result since according to manufacturer's instructions a weak band is interpreted as positive result.

## Discussion

According to ASSURED criteria, the ideal rapid test is A = affordable, S = sensitive, S = specific, U = user friendly (simple to perform in a few steps with minimal training), R = robust and rapid, E = equipment free or minimal equipment, and D = deliverable to those who need them.^22^ We tested the temperature robustness of one brand of dengue RDT and found that despite high temperatures in incubators and at high ambient tropical temperatures in a hut, no changes in test results were noted during the 2-year study. The two readers gave the same interpretations, and there were no invalid tests, no false positive test results, or any obvious damage to the RDT strips.

It is difficult to predict RDT high temperature stability since evaluations of similar immunochromatographic assays, based on detection of Ag and Ab using precoated proteins and migration on nitrocellulose, have given variable results from robustness at high temperature to complete test failure. Indeed, Learmonth and others^20^ reported that six of seven HIV Ab RDT kits were relatively robust despite exposure to higher than recommended temperatures. However, they observed traces of reactivity and atypical discoloration in the negative samples. Chiodini and others^15^ demonstrated that *Plasmodium falciparum* histidine rich protein-2 Ag-based tests maintained sensitivity while plasmodium lactate dehydrogenase (pLDH)-based tests had lower sensitivity after being exposed to high temperatures. Moreover, they showed that the strips themselves were affected after a few days at 45°C with some warping, developement of spots on the nitrocellulose, impairment of blood absorption and flow, and some control line failures. Although Blacksell and others^21^ evaluated eight commercial dengue RDTs, most of them, except for GlobaleMed RDTs, lost their performance after being in a 35°C incubator within 20 and 90 days including the dengue IgM/IgG manufactured by SD. In contrast, the dengue Ag/Ab RDT evaluated by us seems to be more robust, in the face of high temperatures, than the previous generation of dengue RDTs and malaria pLDH Ag RDTs. The different results that we observed in our study in comparison to the study of Blacksell and others may be due to the developments made by the manufacturer over the past 10 years, such as improvement in the stability of the RDT antigen or nitrocellulose media.

The causes of the variability in RDT performances after heat exposure could have various origins. It is expected that proteins will be denatured at high temperature, preventing Ag/Ab binding. However, the resistance of proteins to high temperature varies according to protein sequence and structure, potentially explaining differences in stability observed for different Ag/Ab combinations. Degradation of the strip itself could also be due to the quality of materials used.

The hut used in this study was built to mimic the storage condition in the field, in rural Laos. The temperature recorded exceeded 40°C in March, April, May and June and was higher than that recommended by the RDT manufacturer for approximately 29% of the temperature records during the 2-year study period. The study was performed in Vientiane, in the central region of the country, but it may be expected that the temperatures within houses would be higher in the south. However, the 90-day storage at 45°C and the 2-day storage at 60°C suggest that short-term extreme temperatures do not affect the dengue RDT results. We do not present humidity data as the RDTs were packed in sealed packets, all of which were intact.

The main limitation of our study was that we used only one control serum for each test, NS1, IgM, and IgG, and not a panel of diverse patient sera. Nevertheless, the use of panel of patient sera was not feasible according to the relatively large number of time points tested for the five temperature conditions that required a large serum volume and high number of RDT devices. However, before starting the study, we diluted the different sera to select those just above the limit of detection of the RDT. Our study demonstrated the overall robustness of the RDT after extended exposure at high temperature. Future complementary experiments would be to investigate changes in sensitivity and specificity using a panel of sera tested by RDT after extended storage at tropical room temperature in the field and in parallel by RDT stored according to the manufacturer's instructions. In addition, the tests were performed and interpreted by trained laboratory technicians and not by inexperienced health volunteers and we evaluated only one lot. The SD dengue RDT has a shelf life from manufacture to expiry of 2 years. Although the last testing of RDTs from the hut and 35°C incubation was performed 5 months after the expiry date, we did not find any deterioration of performance. Therefore, this study has demonstrated that the SD dengue RDTs maintains effective diagnostic capacity after extended periods of exposure to heat and even beyond the recommended expiry date. However, although Lao is hot, there are clearly hotter locations, suggesting that this study should be repeated in more extreme hot weather conditions.

An additional issue not investigated here is the high temperatures that RDTs may encounter during transportation, such as on runways and in transport vehicles.^18^,^23^ There is a large diversity of dengue RDTs (∼20) on the market and we have evaluated only one dengue RDT.^21^ Independent assessment of all dengue RDTs should be performed, as has been recommended for malaria RDTs.^17^

The results of this study are not intended to encourage people to use RDTs after they are exposed to temperatures outside the manufacturer's recommendations but it suggests that if such exposure is unavoidable, they can be used with no major changes in diagnostic accuracy. If RDTs have evidence for temperature degradation, positive control well systems, as developed for malaria RDTs, would allow quality control.^24^

With a rapidly increasing number of RDTs for diverse diseases but extraordinary little regulation, there is a need for independent evaluation not just of diagnostic accuracy but of the robustness of such diagnostic accuracy through time in high temperatures.^21^ Although there are temperature stability guidelines for in vitro diagnostics, we have not found any specific guidelines for the evaluation of the temperature stability of RDTs.^25^–^27^ The extensive work on the thermal stability of malaria RDTs, as well as the work of Bienek and others,^28^,^29^ exposing lateral flow assay and blood typing kits to various temperatures and relative humidity conditions, could be adapted to draft such guidelines for evaluation of RDTs in general. With the increasing importance for public health of RDTs, more research on optimizing their field stability is needed, so that their limitations and appropriate use can be better defined.

## Figures and Tables

**Table 1:** Distribution of the number of records in five groups of hut temperatures

Recorded temperature	Number of times the temperature was recorded (percentage) *N* = 25,766
≤ 20°C	970 (3.76)
20.01–25.00°C	4,071 (15.8)
25.01–30.00°C	13,327 (51.72)
30.01–35.00°C	5,677 (22.03)
35.01–40°C	1,569 (6.09)
> 40°C	152 (0.59)

**Table 2:** RDT results of the four control sera tested at different time and temperature

Storage condition	Observations consistent with initial result (percentage)			
	Control sera			
	NS1	IgM	IgG	Negative
Refrigerator 4°C (for 2 years)	30/30 (100)	30/30 (100)	30/30 (100)	30/30 (100)
Incubator 60°C (for 2 days)	2/2 (100)	2/2 (100)	2/2 (100)	2/2 (100)
Incubator 45°C (for 3 months)	8/8 (100)	8/8 (100)	8/8 (100)	8/8 (100)
Incubator 35°C (for 2 years)	24/24 (100)	24/24 (100)	24/24 (100)	24/24 (100)
Hut (for 2 years)	24/24 (100)	24/24 (100)	24/24 (100)	24/24 (100)
Total	88	88	88	88

Ig = immunoglobulin NS1 = nonstructural protein 1; RDT = rapid diagnostic test.
